# Declining trend of HTLV-1 among organ/ tissue donors in Iranian Tissue Bank between 2014–2021

**DOI:** 10.1186/s12977-024-00656-9

**Published:** 2024-12-17

**Authors:** Arash Letafati, Sayed Hamidreza Mozhgani, Mehdi Norouzi, Amir Aboofazeli, Zahra Taghiabadi, Negar Zafarian, Saba Seyedi, Elnaz Mohammad Jaberi, Sedigheh Poursaleh, Maryam Karami, Sheida Sarrafzadeh, Ahmadreza Sadeghi

**Affiliations:** 1 Department of Virology, School of Public Health, Tehran University of Medical Sciences, Tehran, Iran; 2 Research Center for Clinical Virology, Tehran University of Medical Science, Tehran, Iran; 3 Department of Microbiology and Virology, School of Public Health, Alborz University of Medical Sciences, Alborz, Iran; 4 Iranian Tissue Bank and Research Center, Tehran University of Medical Sciences, Tehran, Iran

**Keywords:** Organ transplantation, HTLV-1, Tissue, Brain death, Circulatory death

## Abstract

**Background:**

Human T-cell Lymphotropic Virus type-1 (HTLV-1) infection is associated with serious disorders, including Adult T-cell Leukemia/Lymphoma (ATLL) and HTLV-1–associated myelopathy/tropical spastic paraparesis (HAM/TSP). In addition to sexual, vertical, parenteral, and blood transfusion, organ/tissue transplantation is considered as a transmission route of HTLV infection. Given the substantial risk of HTLV-1 transmission and the subsequent development of HAM/TSP (approximately 40%) in kidney transplant recipients, pre-transplant donor screening is crucial. The present study aimed to investigate the prevalence of HTLV-1 in potential organ/tissue donors referred to the Iranian Tissue Bank and Research Center (ITBRC).

**Materials and methods:**

The study population was potential organ and/or tissue donors referred to ITBRC between 2014 and 2021, including two groups of brain death (potential donors of organs and/or tissues) and circulatory death donors (potential tissue donors from Iranian Legal Medicine Organization). Initial screening was performed using enzyme-linked immunosorbent assay (ELISA), and positive cases were confirmed for HTLV-1 infection with polymerase chain reaction (PCR).

**Results:**

111 out of 3,814 donors were positive for HTLV-1 (3%). The rate of positive tests between 2014 and 2017 was 6%, which was significantly higher than the positive tests percentage between 2017 and 2021 with 0.5% (*P*-value < 0.001). The rate of test positivity in females was 4% compared to 2% in males (*P*-value = 0.001). Furthermore, individuals diagnosed with brain death exhibited a significantly lower likelihood of HTLV-1 infection (0.2%) compared to cases with circulatory death (4%) (*P*-value < 0.001).

**Conclusion:**

Considering the contraindication of organ/tissue donation from donors with HTLV-1 positive test, these findings give an insight into the prevalence of HTLV-1 among potential organ/tissue donors in Iran. Moreover, the higher prevalence of HTLV-1 infection in circulatory death donors from Iranian Legal Medicine Organization urges for cautious evaluation in these donors.

## Introduction

Human T-cell Lymphotropic Virus type-1 (HTLV-1) is correlated with severe disorders like Adult T-cell Leukemia/Lymphoma (ATLL) and HTLV-1-associated myelopathy/tropical spastic paraparesis (HAM/TSP). HTLV-1 infection effects millions of world population, but treatment options remain limited [1, 2]. HTLV-1 infects CD4 + T-lymphocytes through de novo infection (facilitated by viral cell-to-cell transmission) which is followed by clonal expansion (cell proliferation within infected clones) [3, 4]. The progression of HTLV-1 towards cancer results in increased T-cell activation and reshaping infected cells to T regulatory (Treg) subset. These modifications facilitates virus immune escape and prolongs the survival of infected cells, leading to ATLL [5]. It is estimated that approximately 5% of HTLV-1 infections result in ATLL [6, 7]. Additionally, HTLV-1 induces HAM/TSP, an infrequent inflammation of the spinal cord characterized by progressive neurological manifestation such as spastic paraparesis and neurogenic bladder as well as sensory abnormalities in the lower limbs [8]. The demyelinating occurred due to HAM/TSP is similar to primary progressive multiple sclerosis (MS) and immune cells are central to the development of these demyelinating diseases [9].

The likelihood of developing HAM/TSP among individuals infected with HTLV-1 differs based on ethnicity. For instance, Japanese individuals with HTLV-1 have a lower risk of developing HAM/TSP, estimated lifetime incidence at around 0.25% (3.1 × 10^− 5^ cases/year), compared to Caribbean individuals with HTLV-1, who have a higher risk at approximately 1.9% [10, 11]. A three-year study conducted in western Iran identified 11 cases of HTLV-1-associated HAM/TSP among all spastic paraparetic patients examined [12]. The worldwide prevalence of HTLV-1 infection is estimated to be approximately 5 to 10 million people [13]. Moreover, people living with HTLV-1 exhibited a significantly elevated adjusted risk of death, with relative risks of 1.57 compared to individuals negative for HTLV-1 [14].

HTLV-1 can be transmitted through sexual, vertical, parenteral (contaminated blood), and organ transplantation [15–17]. Accordingly, some countries use HTLV antibody screening for blood donors [18, 19]. HTLV-1 transmission with transplantation might be associated with faster progression to HAM/TSP [20]. A study in Japan which is endemic for HTLV-1, reported that HTLV-1 transmission through kidney transplantation poses a significant risk with a 40% chance of developing HAM/TSP [21]. Given the increased risk of HTLV-1-related diseases in immunocompromised individuals, donors and recipients screening is highly recommended [19]. Regarding the significance of safe organ/tissue donation, we aimed to investigate the prevalence of HTLV-1 among potential deceased donors referred to Iranian Tissue Bank and Research Center (ITBRC).

## Materials and methods

### Study Population and Sample Collection

The study encompassed 3814 potential deceased donors (2,577 male and 1,237 female) referred to ITBRC between 2014 and 2021. Potential donors were divided in two groups of brain death donors and those with circulatory death referred from Iranian Legal Medicine Organization (IRLMO). Blood samples were taken from donors under the explicit informed consent of each donor’s next-of-kin prior to donation. The study was conducted according to Ethical approval Code (IR.TUMS.SPH.REC.1402.329) issued by the Tehran University of Medical Sciences. The median age of the participants in the study was 32, the youngest was two years old and the oldest was 60.

## ELISA assessment and PCR test

Serum samples were tested for anti-HTLV-1 and HTLV-2 antibodies based on the enzyme-linked immunosorbent assay (ELISA) by using ELISA kits (DIA PRO, Italy) according to the manufacturer’s instructions. Samples with a sample/cut-off ratio below 0.9 were considered to be negative and values exceeding this threshold indicated as HTLV-1 and − 2 reactivity. RT-qPCR test was performed targeting the TAX and HBZ genes to validate the HTLV-1 seropositive samples. Additionally, the primer sequences of these two genes were chosen according to prior research [22].

### Statistical analysis

The statistical analysis was conducted using Stata-version 17 software. Descriptive statistics were used to summarize the distribution of HTLV-1 test results by year of sampling, gender, and donor type. The chi-square test was used to evaluate the significance of differences in infection rates across demographic subgroups.

## Result

The analysis of HTLV-1 infection trends over time and gender distribution showed a significant change in infection rates over time, with a decreasing trend between 2017 and 2021. Table 1 shows the distribution of study population and the number and percentage of donors with HTLV-1/2 reactivity by year of sampling. In the whole population, 111 donors were HTLV-1 positive (3%) and 3703 revealed to have negative results for HTLV-1 test (97%). The percentages of positive and negative tests for HTLV-1 infection have been presented by year (2014 to 2021). It can be seen from Table 1 that the highest percentage of infected cases among potential donors was recorded in 2014 and the lowest was in 2018. In 2014–2016 the reactivity rate was 6%. compared with 0.5% in 2017 to 2021 (P-value < 0.001) (See Table 2).

**Table 1:** Frequency and percentage of HTLV-1/2 reactivity results by year

	2014	2015	2016	2017	2018	2019	2020	2021	Total
Negative	530 (92%)	591 (96%)	499 (94%)	411 (99%)	501 (100%)	372 (100%)	410 (99%)	389 (99%)	3703 (97%)
Positive	43 (8%)	25 (4%)	33 (6%)	2 (0.5%)	0 (0%)	1 (0%)	2 (0.5%)	5 (1%)	111 (3%)
Total number of tests per year (%)	573 (15%)	616 (16%)	532 (14%)	413 (11%)	501 (13%)	373 (10%)	412 (11%)	394 (10%)	3,814

**Table 2:** Frequency and percentage of HTLV-1 reactivity results in two period

	Negative	Positive	Total
2014–2016	1,620	101 (6%)	1,721
2017–2021	2,083	10 (0.5%)	2,093

Furthermore, a gender-based difference in infection rates was detected, with women having a higher prevalence of HTLV-1 infection compared to men. The distribution of people on gender basis is shown in Table 3. In the subgroup of women, 1158 individuals were tested negative for HTLV-1 infection and 52 (4%) were tested positive. This was significantly higher than in male individuals with 2% of positive tests (P-Value = 0.001). Finally, in comparison according to type of death, the percentage of HTLV-1 infection among brain death donors was 0.2% compared to 4% in donors with circulatory death (P-Value < 0.001).

**Table 3:** Frequency and percentage of HTLV-1 reactivity results by gender and donor type

	Negative	Positive	Total
Female	1185 (96%)	52 (4%)	1,237 (32%)
Male	2,518 (98%)	59 (2%)	2,577 (68%)
Total	3,703 (97%)	111 (3%)	3,814
Brain death	1,299 (99.8%)	2 (0.2%)	1,301
Circulatory death	2,404 (96%)	109 (4%)	2,513

## Discussion

The global distribution of HTLV-1 shows higher rates in certain regions such as Japan and northeastern of Iran in Asia, Central Australia, South America, the Caribbean Islands, as well as the western and central areas of Africa, and Romania in Europe [15, 16]. In African regions heavily affected by HTLV-1, the prevalence of infection has been estimated 0.3-3% [23]. In Central and South America & the Caribbean, known as endemic regions for HTLV-1, 1.3% of pregnant women were found to have HTLV-1/2 infection, with the majority of cases being HTLV-1 [24]. A study conducted in 2020–2021 in Japan on individuals donating blood for the first time estimated the number of HTLV carriers at about 534,000 [25]. In Iran the overall prevalence ranges from 0.07 to 1.8%. However, in endemic regions such as Khorasan provinces in northeast Iran, the prevalence is 2.5% [26]. In Mashhad the capital city of the Khorasan province the prevalence of HTLV-I infection was 2.12% [27]. In in Neyshabur another city in Khorasan province the HTLV-1 prevalence was 3.6% [28]. In a research study conducted in Torbat-e Heydarieh, a significant city in Khorasan province, the sero-reactivity for HTLV-1 among the participants was found to be 2%, with an actual prevalence of 1.25% showing the presence of HTLV-1 provirus in lymphocytes [29]. However, in another geographical location in Iran such as Alborz province the HTLV-1 positivity rate was 0.13% [30] and in Golestan province was approximately 0.09% [31].

According to our findings, 3% of potential organ/tissue donors between 2014 and 2021 were found to be infected with HTLV-1. This HTLV-1 prevalence in 2021 was 1%. Similarly, during 2016–2017, a study conducted in Tehran, evaluating the seroprevalence of HTLV-1 among 139 kidney transplant recipients, reported a prevalence rate of 0.71% [32]. A 2018 cross-sectional study found that the prevalence of anti-HTLV-1/2 antibodies among blood donors was 1.7%, with 0.05% showing reactivity specifically to HTLV-1 [33]. Moreover, in a study conducted in Urmia, including 91 kidney transplant recipients, HTLV-1 antibody detection using ELISA revealed only one positive case. This finding was subsequently confirmed through Western blot analysis [34]. The results of a study conducted in Colombia between 2010 and 2017, the seroprevalence of HTLV-1 among organ donors was found to be 0.2% [35]. In a Spanish study on 5751 organ transplant donors/recipients, initial screening identified 9 cases (6 donors and 3 recipients) seropositive cases; however, further evaluations with Western blot and PCR test confirmed HTLV-1 infection in only two of the donors [19]. Moreover, in Japan, 26 out of 329 cases who underwent living donor liver transplantation (7.9%) were HTLV-I carrier [36].

Analysis of HTLV-1 infection trends in our study revealed a decreasing trend after 2017. Likewise, a study of HTLV-1/2 in blood donors of Iran revealed a declining prevalence of HTLV-1/2 from 2010 to 2018 [37]. The observed decline in HTLV-1 infection rates suggests a potential impact of preventive measures or changing epidemiological dynamics. Gender-based analysis demonstrated that women exhibit a greater incidence of infection compared to males. The higher rate of infection among women warrants further investigation into gender-specific risk factors and healthcare disparities. However, in a longitudinal investigation between 2002 and 2007 in Iran involving 1548 tissue donor cases, the seroprevalence of HTLV-1 was found to be 1.61% (*n* = 25), with a higher incidence among male donors compared to the female donors [38].

Furthermore, we found that in a comparison between brain-dead donors and circulatory death donors, the former group exhibited a significantly lower likelihood of HTLV-1 infection (0.2%) compared to the latter (4%). This can also be correlated with the fact that mainly, homeless people are referred to us which may not reflect general health status. Another research conducted in Tehran, reported 3.4% of the 54 brain-dead organ donors positive for anti-HTLV-1 antibodies testing with ELISA. However, further verification through Western blotting showed no case of HTLV-1 positive results [39].

Transplantation of HTLV-1 infected graft can be dangerous for the recipient [40]. The high transmission capability of HTLV-1 and the secondary immunodeficiency due to intensive immunosuppression pose the recipients in a considerable risk of severe HTLV-1 infection and its complications [41]. One study reported three cases of HAM/TSP in liver and kidney transplant recipients, occurring 2 years after transplantation from an asymptomatic donor [42]. Furthermore, one investigation in 89 renal transplants performed between 2006 and 2021 detected four HTLV-1-positive. Two recipients, who tested positive for HTLV-1 infection, progressed to ATL approximately 3 years post-transplant [43]. The unpredictable outcomes of HTLV-1 infection acquired via transplantation could lead to severe illness. To avoid adverse effects of transplantation from infected organs, it is recommended not to use them, especially in non-imminently life-threatening situations like renal transplant [44].

Despite our efforts, there have been some limitations. First, the present study focused on organ and/or tissue donors, which may restrict the generalizability of findings to general population. Additionally, the absence of investigation into the prevalence of HTLV-1 infection among living organ donors presents a notable gap in our knowledge of total transmission risk in recipients. Also, the reduction of this prevalence situation may be related to circulatory failures that a double check for HTLV-1 and HTLV-2 was done in forensic medicine and negative samples were sent to us in terms of serology. However, the negative result of PCR was a confirmation of the absence of infection, and this test was performed in our center. Further research is required to offer a more detailed insight into HTLV-1 infection in solid organ transplantation.

## Conclusion

Despite regional variations in HTLV-1 prevalence across the globe, our findings indicated a notable presence of HTLV-1 infection among our study group in Iran, with a decreasing trend in recent years. Gender-based analysis suggests higher prevalence in women warranting additional investigations into gender-specific risk factors. Moreover, the association between donor type and HTLV-1 infection risk underscores the importance of donor selection in transplantation. Given the significant risk associated with HTLV-1 transmission from donors to organ recipients, it is essential to continue monitoring HTLV-1 prevalence among allograft donors, implementing robust screening protocols to reduce transmission risks.

## Data Availability

No datasets were generated or analysed during the current study.
